# Assessing Choroidal Thickness in Pediatric Patients With Unilateral Strabismic Amblyopia by Using Spectral Domain-Enhanced Depth Imaging-Optical Coherence Tomography

**DOI:** 10.7759/cureus.60219

**Published:** 2024-05-13

**Authors:** Cem Evereklioglu, Ayşe Merve Keskin, Hatice Kübra Sönmez, Hatice Arda

**Affiliations:** 1 Division of Pediatric Ophthalmology & Strabismus, Department of Ophthalmology, Erciyes University Medical Faculty, Kayseri, TUR; 2 Department of Ophthalmology, Moorfields Eye Hospital, London, GBR

**Keywords:** strabismus, optic coherence tomography, oct, choroidal thickness, amblyopia

## Abstract

Objective

In this study, we aimed to evaluate the choroidal thickness in patients with unilateral strabismic amblyopia by using spectral domain-enhanced depth imaging-optical coherence tomography (SD-EDI-OCT) (Heidelberg Engineering GmbH, Heidelberg, Germany).

Methods

Twenty-five children with strabismic amblyopia and 20 age- and sex-matched healthy controls were included in this study. Seven sections were obtained, each comprising 25 repetitive images from each section at 200-micron intervals, and measurements were taken from nine different points at vertical and horizontal lines (1 and 3 mm from the subfoveal, superior, inferior, temporal, and nasal regions), centered on the fovea, using SD-EDI-OCT. Choroidal thickness values were obtained by measuring the distance between the basal border of the retinal pigment epithelium and the choroidoscleral border. The Mann-Whitney U test was used to compare choroidal thickness between the amblyopic and the control groups.

Results

The mean age of children with amblyopia and that of controls were 8.4 ±2.7 and 9.9 ±3.3 years, respectively (p=0.120). The mean subfoveal choroidal thickness was 372.8 ±78.9 μm in amblyopic eyes and 372.4 ±79.3 μm in the fellow eyes, both of which were thicker than the control eyes (310.9 ±76.3 μm; p<0.05 for each). Similarly, the mean values for the choroidal thickness of the amblyopic children at 1 mm nasal (320 ±86 μm), 1 mm superior (363 ±70 μm), and 3 mm superior (336 ±62 μm) were also significantly thicker than those of the corresponding control eyes (p<0.05 for each). There was a negative correlation between the subfoveal choroidal thickness and axial length (r=-0.332, p=0.005). There were no correlations between the choroidal thickness, age, and visual acuity.

Conclusions

The choroidal thicknesses of strabismic and fellow eyes were similar in patients with strabismic amblyopia. However, the choroidal thickness of both eyes in strabismic children was significantly thicker than those of the healthy controls. Emmetropization may be defective in both eyes of strabismic amblyopic patients.

## Introduction

Amblyopia is characterized by a unilateral or bilateral decrease in visual acuity without any other ocular pathology. It develops due to abnormal visual experiences in the early stages of life. There are mainly three types of amblyopia: strabismic, refractive, and visual deprivation; empirical evidence reveals that amblyopia develops due to changes in the visual cortex [1]. However, many studies have been conducted to investigate the presence of subclinical abnormalities in the anterior striate cortex despite the normal globe structure in amblyopic eyes [2,3]. Although it has been shown that the lateral geniculate nucleus (LGN) and the visual cortex are primarily affected in amblyopia, possible changes in the ocular glob still arouse curiosity [4]. A few previous studies have alluded to potential secondary changes in the retina [5,6].

With the advent of optical coherence tomography (OCT), retinal changes in amblyopia have also been investigated. Szigeti et al. found minimal changes in the outer nuclear layer in their study on unilateral amblyopia [5]. On the other hand, Altintas et al. showed no difference between the healthy population and children with strabismic amblyopia regarding the thicknesses of the retinal nerve fiber layer, macular thickness, and macular volume in [7].

The choroid provides vascular support to the outer retinal layer and is responsible for ocular temperature, intraocular pressure regulation, and light absorption [8]. In addition, it has been shown that it affects emmetropization [9]. The development of enhanced depth imaging-OCT has enabled us to investigate the thickness of the choroid in detail, which might be affected by many diseases [10]. The first study in the literature on choroidal thickness was performed by Nishi et al. on children with anisometropic amblyopia. They demonstrated that there was a significant difference in choroidal thicknesses between amblyopic eyes, fellow eyes, and the control group, which was correlated with the axial length [11]. Subsequent studies have also focused on the anisometropic group, and few studies have included strabismic patients [12,13,14,15]. This study aimed to assess the effect of amblyopia on the choroidal thickness.

## Materials and methods

This cross-sectional, non-interventional study was conducted at the Division of Pediatric Ophthalmology & Strabismus, Department of Ophthalmology, Erciyes University Medical Faculty in Kayseri, Türkiye. The study adhered to the tenets of the Declaration of Helsinki, it was approved by the Erciyes University Institutional Review Board (approval number: 2023/458). Consent was obtained from the parents of the children included in this study to perform the measurements and to review their medical records.

A total of 25 children with strabismic amblyopia and 20 age- and sex-matched healthy control subjects were included in the present study. All pediatric patients and controls underwent a full ophthalmologic examination and the visual acuity was evaluated with the Snellen chart. Refractive status was measured half an hour after a cycloplegic agent was instilled three times in five-minute intervals. In addition to the ophthalmological examination, axial length measurements (Nidek Optical Biometer AL-Scan device, Nidek Inc., San Jose, CA) and enhanced depth imaging-optical coherence tomography (EDI-OCT) were performed.

The inclusion criteria were as follows: patients with a best-corrected visual acuity (BCVA) below 0.8 with a 2-line difference between the fellow eye; refractive error of less than the 1D difference between the eyes; and heterotropia of more than 10 prism diopters. The exclusion criteria were as follows: eccentric fixation; any ocular and systemic diseases; and decreased visual acuity due to retinochoroidal diseases. Only the right eyes of emmetropic children were enrolled in the control group. It was ensured that they had normal visual acuity with no ocular or systemic diseases.

**Figure 1:**
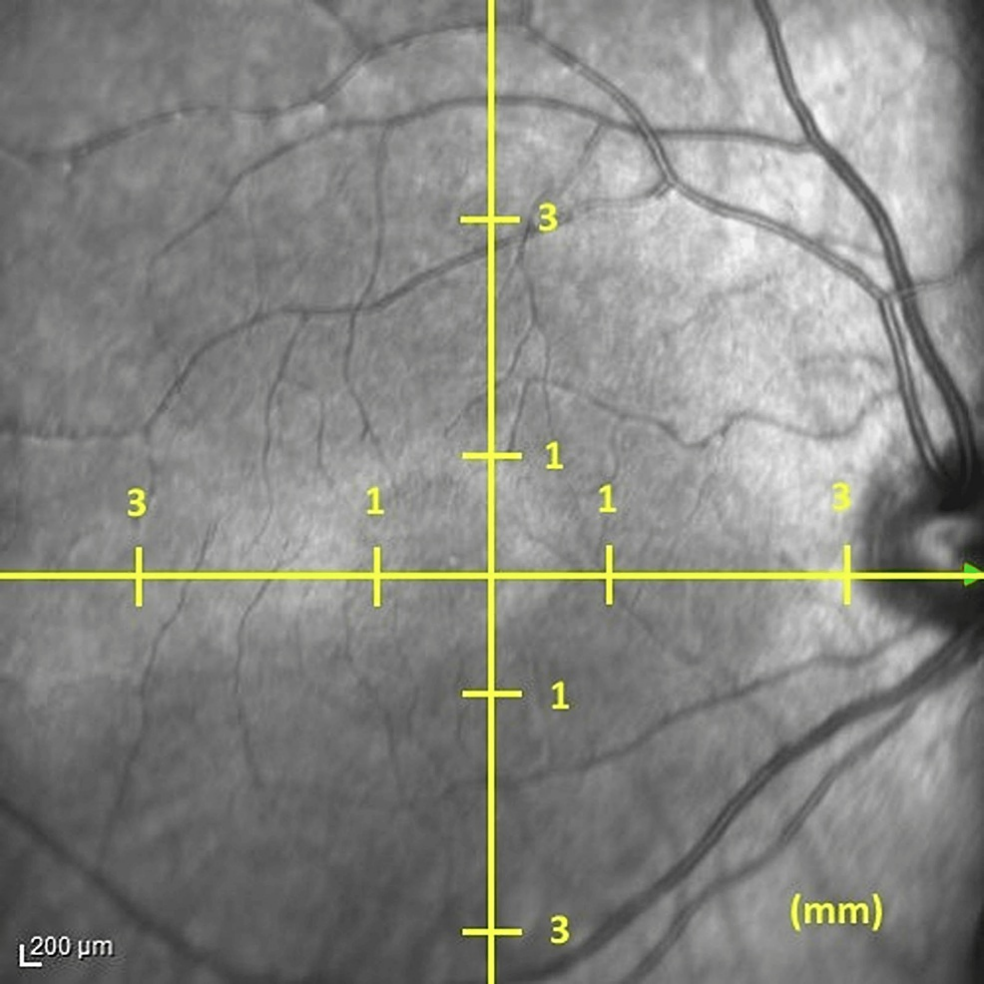
Choroidal thickness measurement Nine different points for choroidal thickness measurements taken from the subfoveal region and the 1 mm and 3 mm superior, inferior, temporal, and nasal aspects of the fovea

Spectral domain EDI-OCT (SD-EDI-OCT) imaging was performed by the same experienced technicians using a Heidelberg machine (Heidelberg Engineering, GmbH, Heidelberg, Germany). Seven sections were taken, each comprising 25 repetitive images from each section at 200-micron intervals, vertical and horizontal, centered on the fovea. Measurements were taken between 10 and 12 o'clock in the morning to reduce the risk of diurnal variation. The measurements of the choroidal thickness were taken by two different practitioners at different times. The result was recorded and the average of the two measurements was obtained. Choroidal thickness values were obtained by measuring the distance between the basal border of the retinal pigment epithelium and the choroidoscleral border. Measurements were taken from nine different points, 1 mm and 3 mm from the subfoveal, superior, inferior, temporal, and nasal regions (Figure 1).

Statistical analysis

Regarding descriptive statistics of the data, mean, standard deviation (SD), median, minimum, maximum, frequency, and ratio values were used. The distribution of variables was measured with the Kolmogorov-Smirnov test. ANOVA (Tukey test), Kruskal-Wallis, Mann-Whitney U test, and independent sample t-test were used in the analysis of visual acuity, refraction value, axial length, and choroidal thickness data. The chi-square test was used in the analysis of age and gender data. Spearman correlation analysis was used in the correlation analysis of choroidal thickness with age, visual acuity, refraction value, and axial length. All statistical analyses were performed using SPSS Statistics version 22.0 (IBM Corp., Armonk, NY).

## Results

The mean age of the strabismic group was 8.4 ±2.7 (range: 5-16) years whereas it was 9.9 ±3.3 (range: 5-15) years in the control group (p>0.05) (Table 1).

**Table 1:** Demographic characteristics of the groups SD: standard deviation

		Amblyopic eye	Controls	P-value
Age, years, mean ±SD (range)		8.4 ±2.7 (5–16)	9.9 ±3.3 (5–15)	0.120
Gender, n (%)	Female	12 (48)	8 (60)	0.592
	Male	13 (52)	12 (40)	

The mean visual acuity [logarithm of the minimum angle of resolution (logMAR)] was significantly decreased in the amblyopic group (0.5 ±0.2) when compared with fellow eyes (1.0 ±0.1) and controls (1.0 ±0.0). The axial lengths were similar between the amblyopic eyes (22.0 ±0.8 mm) and fellow eyes (22.0 ±0.8 mm), both of which were lower than control eyes (23.3 ±0.9 mm). The mean value of the spherical equivalent was 2.3 ±2.0 D in amblyopic eyes, 2.3 ±1.9 D in fellow eyes, and 0.4 ±0.8 D in control eyes (Table 2).

**Table 2:** Comparison of BCVA, axial length, and spherical equivalent values between the groups ^*^The difference between the amblyopic eye and controls. ^ǂ^The difference between the fellow eye and controls BCVA: best-corrected visual acuity; SD: standard deviation

	Amblyopic eye	Fellow eye	Control	P-value
BCVA, mean ±SD (range)	0.5 ±0.2 (0.1–0.8)	1.0 ±0.1 (0.8–1.0)	1.0 ±0.0^*^ (1.0–1.0)	<0.001
Axial length, mm, mean ±SD (range)	22.0 ±0.9 (20.02–23.6)	22.0 ±0.8 (20.4–23.3)	23.3 ±0.9^*ǂ^ (22.1–25.5)	<0.001
Spherical equivalent, D, mean ±SD (range)	2.3 ±2.0 (-1.0–6,8)	2.3 ±1.9 (-0.5–7.0)	0.4 ±0.8^*ǂ^ (-0.8–2.0)	<0.001

The thickest part of the choroid was at the subfoveal choroidal location and the thinnest area was at the 3 mm nasal to the fovea among both amblyopic and control eyes (Table 3). Although the mean subfoveal choroidal thickness was similar between amblyopic eyes (372.8 ±78.9 μm) and fellow eyes (372.4 ±79.3 μm), both values for amblyopic children were significantly higher than those found in the same locations of the control eyes (310.9 ±76.3 μm, p<0.001). In addition, measurements at 1 and 3 mm superior locations as well as at 1 mm nasal positions were found to be significantly higher when compared with those found in the same positions (p<0.05 for each). On the other hand, all other comparisons were found to be similar (p>0.05 for each).

**Table 3:** Choroidal thicknesses in the amblyopic eyes, fellow eyes, and control eyes ^*^The difference between the amblyopic eyes and controls. ^ǂ^The difference between the fellow eyes and controls SFCT: subfoveal choroidal thickness; SD: standard deviation

	Amblyopic eyes, μm, mean ±SD (range)	Fellow eyes, μm, mean ±SD (range)	Controls, μm, mean ±SD (range)	P-value
SFCT	372.8 ±78.9 (197–509)	372.4 ±79.3 (231–521)	310.9 ±76.3^*ǂ^ (207–487)	0.015
1 mm superior	363 ±70 (222–465)	362 ±75 (214–513)	305 ±68^*ǂ^ (205–466)	0.013
3 mm superior	336 ±62 (218–465)	330 ±66 (228–481)	289 ±59^*ǂ^ (203–429)	0.034
1 mm inferior	333 ±56 (217–434)	333 ±61 (215–456)	291 ±72 (160–458)	0.051
3 mm inferior	282 ±47 (160–352)	284 ±41 (201–351)	266 ±61 (158–378)	0.458
1 mm temporal	333 ±91 (197–462)	349 ±71 (220–462)	295 ±71 (169–407)	0.072
3 mm temporal	309 ±78 (146–444)	312 ±70 (186–421)	275 ±68 (152–399)	0.178
1 mm nasal	320 ±86 (147–485)	325 ±76 (197–505)	256 ±82^*ǂ^ (152–440)	0.011
3 mm nasal	177 ±71 (70–341)	165 ±72 (68–370)	160 ±56 (88–305)	0.664

Similar results were found for the measurements at 1 and 3 mm superior locations as well as at 1 mm nasal position (p<0.05 for each). All other comparisons were found to be similar (p>0.05 for each).

There was no significant correlation between the visual acuity and choroidal thickness at all measurement locations (p>0.05 for each). However, there was a significant negative correlation between the axial length and subfoveal choroidal thickness, 1 mm nasal, 3 mm nasal, 1 mm temporal, 1 mm superior, 3 mm superior, and 1 mm inferior measurements of the choroid (p<0.05 for each). There was a significant (p<0.05 for each) positive correlation between spherical equivalent value and subfoveal choroidal thickness, 1 and 3 mm nasal, 1 and 3 mm superior, and 1 mm inferior (Table 4). On the other hand, there was no significant correlation between age and choroidal thickness at all measurement locations (p>0.05 for each).

**Table 4:** Correlation of choroidal thickness with visual acuity, axial length, and refractive error BCVA: best-corrected visual acuity; SFCT: subfoveal choroidal thickness

Spearman correlation analysis		BCVA	Axial length	Spherical equivalent	Age
SFCT	r	-0.209	-0.332	0.304	0.126
	p	0.083	0.005	0.011	0.410
1 mm nasal	r	-0.162	-0.351	0.306	0.101
	p	0.182	0.003	0.010	0.509
3 mm nasal	r	-0.105	-0.300	0.244	0.059
	p	0.386	0.012	0.042	0.702
1 mm temporal	r	-0.162	-0.247	0.184	0.253
	p	0.180	0.040	0.128	0.094
3 mm temporal	r	-0.116	-0.210	0.178	0.106
	p	0.341	0.081	0.140	0.487
1 mm superior	r	-0.234	-0.267	0.301	0.084
	p	0.051	0.025	0.011	0.582
3 mm superior	r	-0.235	-0.355	0.392	-0.024
	p	0.050	0.005	0.001	0.877
1 mm inferior	r	-0.195	-0.256	0.257	0.151
	p	0.105	0.026	0.032	0.322
3 mm inferior	r	-0.079	-0.181	0.178	0.245
	p	0.516	0.134	0.141	0.104

**Figure 2:**
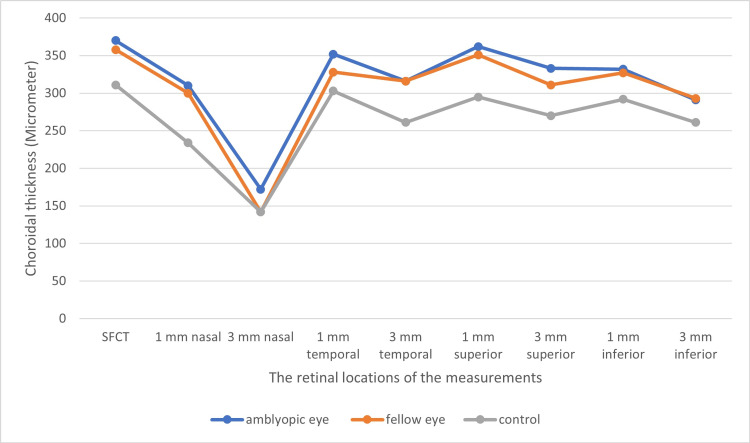
Comparison of choroidal thicknesses between groups SFCT: subfoveal choroidal thickness

Regarding the choroidal thickness profile in amblyopic eyes, fellow eyes, and control eyes, the thickest region of the choroid was the subfoveal region in all three groups, which was followed by 1 mm superior to the fovea (Figure 2). When we checked the figure, the mean subfoveal choroidal thicknesses in both eyes of amblyopic children were higher than the same locations of control eyes. Similar findings were seen in the superior locations at 1 and 3 mm and the nasal location at 1 mm.

## Discussion

The present study found that subfoveal choroidal thickness and some other parts of the choroid measurements of the amblyopic and fellow eyes were significantly higher than the corresponding points of the control eyes. We observed that the axial length of the amblyopic eyes and the other eye was significantly shorter than the control eye, which correlated with the choroidal thickness of the groups. Axial length negatively correlated with whole choroidal thickness measurement in all three groups, consistent with the literature [16]. Although there are a few studies about choroidal thickness and amblyopia in the literature, the majority of the reports have evaluated anisometropic patients, and two studies involved strabismic amblyopia. Niyaz et al. have analyzed the choroidal thickness in subtypes of strabismus, anisometropia, and hypermetropia. They found that there was no statistically significant difference between the two eyes in strabismic amblyopia patients, in line with our study [15].

In our study, refractive values were hyperopic in both deviated and fellow eyes. Moreover, there was no difference between the mean spherical equivalent value of the deviated eye and that of the fellow eye. This could be attributed to the defective emmetropization [17]. The refraction values in our study also align with Ingram et al.'s findings [17]. They reported emmetropization to be defective in both eyes of children with heterotropia or microtropia at six months and 42 months, which was found in 80% of the patients, regardless of the spherical value at six months [17]. Nishi et al. stated that hyperopic defocus caused choroidal thinning in the fellow eye of anisometropia patients. They suggested that the choroid was thicker, and the development of the eye was limited since choroidal compensation did not occur in the amblyopic eye [11]. It has been shown that choroidal thickness decreases with age [18,19]. Fujiwara et al. reported that the subfoveal choroidal thickness decreased by 20 µm every 10 years in healthy Japanese individuals. Nonetheless, in this study, both groups showed no significant correlation between choroidal thickness and age, which may be due to the narrow age range in the present study (5-16 years(.

It has also been reported that the thickest region of the choroid can vary in amblyopias. The thickest region of the macular choroid in the amblyopic eye was found to be the subfoveal region, while the thickest region in the intact eye and control eyes was the temporal region [11]. These results were compatible with the choroidal thickness profile in Ruiz Moreno et al.'s study evaluating the choroidal thickness in healthy children [20]. Therefore, it was thought that the choroidal thickness profile in amblyopic eyes differed from normal eyes. However, there was no difference between the amblyopic and fellow eyes regarding the choroidal profile. On the other hand, the thickest part of the choroid was in the subfoveal region in all three groups. The superior choroid was the second thickest part after the subfoveal region, followed by the temporal, inferior, and nasal choroids.

The increase in the metabolic demand of the outer retina is associated with cone photoreceptor density in the fovea and rod photoreceptor density peaking in the central and superior fovea [21]. Consistent with our study, the thicker choroidal thickness in the central and superior regions of the fovea may also be related to the metabolic requirement of the retina. This is because the thickest regions of the choroid are the fovea and superior regions. Most participants of our study group were still on amblyopia treatment as their cortical maturation had not been completed yet. Regarding the effect of treatment on the choroid, previous studies have reported no significant differences in subfoveal choroidal thickness after treatment between the amblyopic and fellow eyes [13,22].

Limitations

This study has a few limitations. Firstly, although no significant correlation was found between age and choroidal thickness, the age range could have been narrower, in line with previous studies. Secondly, the number of study patients was low. Finally, the refractive status and axial length of the study population may have been similar when compared with control groups.

## Conclusions

Increased choroidal thickness bilaterally in each eye suggests that emmetropization may be defective simultaneously in both strabismic and fellow non-strabismic eyes in pediatric patients with strabismic amblyopia. The thicker choroidal thickness in the central and superior foveal regions in the fundus may be linked to the metabolic requirement of the retina as the abovementioned regions are the thickest parts of the choroid. Further studies are required to determine which part of the choroid is affected by amblyopia, based on the evaluation of the choroidal vascularity index.
